# Docking analysis of verteporfin with YAP WW domain

**DOI:** 10.6026/97320630013237

**Published:** 2017-07-31

**Authors:** Ilham Kandoussi, Wiame Lakhlili, Jamal Taoufik, Azeddine Ibrahimi

**Affiliations:** 1 Laboratoire de Biotechnologie (MedBiotech), Faculté de Médecine et de Pharmacie de Rabat, Université Mohammed V Rabat, Morocco; 2 Laboratoire de Chimie thérapeutique Faculté de Médecine et de Pharmacie de Rabat, Université Mohammed V Rabat,Morocco

**Keywords:** Hippo, TEAD, YAP, kinase, vertoporphine, docking

## Abstract

The YAP oncogene is a known cancer target. Therefore, it is of interest to understand the molecular docking interaction of verteporfin (a derivative of benzo-porphyrin) with the WW domain of YAP (clinically used for photo-dynamic therapy in macular degeneration) as a potential WW domain-ligand modulator by inhibition. A homology protein SWISS MODEL of the human YAP protein was constructed to dock (using AutoDock vina) with the PubChem verteporfin structure for interaction analysis. The docking result shows the possibilities of verteporfin interaction with the oncogenic transcription cofactor YAP having WW1 and WW2 domains. Thus, the ability of verteporfin to bind with the YAP WW domain having modulator activity is implied in this analysis.

## Background

The Hippo pathway is known to be involved in cell proliferation, differentiation, growth, cell death [1] as well as the control of organ size and tumorigenesis [2]. Key stakeholders in this pathway have been identified first in Drosophila and later their orthologs in mammals [3]. One of the key players is YAP protein, which acts as transcriptional co-activators for the TAED transcription factor regulating the genes involved in cell proliferation and apoptosis [4,5,6]. However, the deregulation of one of these leads to certain diseases including cancer [7]. The amplification of YAP was detected in breast cancer and hepatocellular carcinoma [8]. Thus, pharmacological inhibition of YAP is considered as an effective anticancer strategy.

Hippo pathway is activated by cell contact [2], which triggers a cascade of interactions leading to the phosphorylation of S YAP various residues including S127. LATS and MST, which are two upstream kinases in the pathway, were shown to be involved in this phosphorylation through PPxY grounds [9,10]. On the other hand, YAP acted in the same mechanism through the WW domains [9,10]. The pS127 and the neighboring residues have binding site for the 14-3-3 proteins, which is responsible for cytoplasmic localization of YAP. In cytoplasm, YAP mediates pro-apoptotic signals [11]. Phosphorylation of the S residue other than S127 leads to the ubiquitination and proteasomal degradation of YAP [2]. The lack of S127 phosphorylation leads to the translocation to the nucleus of the cell and the complex TAED / YAP formation, hence the transcription of the genes for growth and anti-apoptotic genes [12]. Previous studies [10,13] have shown the involvement the PPxY in the formation of functional complexes in order to inhibit YAP by its cytoplasm retention. The interaction between the WW domain of YAP and LATS1 kinase and between the WW domain of YAP and AMOTL1 cell junction protein-to-cell [10,13], which anchors the YAP protein in the cytoplasm, like action of P14-3-3 is important [11].

YAP1 isoform possess a single WW domain and YAP2 possess two WW domains (WW1 and WW2) [4]. The structural models were built and PPxY have shown that peptides bind to the hydrophobic groove of the WW1 domain of β sheet to residues Y188 / T197 / L190 / H192 / Q195 domain. The PPxY peptides were also shown to bind to the domain YAP WW2 involving W258, Y247, T256, I249, H251 and K254 [14]. However, both YAP WW domains act as independent units with different binding preferences [15]. It was predicted that the cardiac glycoside digitoxin has an affinity for the WW domain of dystrophin and has important implications for the design of therapeutic small molecules modulating WW domains [16]. Verteporfin a derivative benzo-porphyrin, clinically used in photodynamic therapy for macular degeneration as through its interaction to WW domain-ligand as a modulator is known [17]. Indeed, using a screen containing drugs approved by the FDA, it was shown that the verteporfin has the ability to disrupt the interaction YAP and TAED leading to cell proliferation reduction by YAP [17].

It is also known that verteporfin have a direct inhibitory effect on the growth of cancer cells without light activation [18], but via the disruption of complex YAP-TEAD and prevention of induced oncogenic growth YAP [18]. Therefore, it is of interest to understand the molecular docking interaction of verteporfin (a derivative of benzo-porphyrin) and clinically used in photodynamic therapy for macular degeneration with the WW domain of YAP.

## Methodology

### Homologous Modeling

The sequence for the human YAP protein target was downloaded from the Uniprot database (www.uniprot.org) for homology modeling using SWISS-MODEL (http://swissmodel.expasy.org /) [19,20]. We chose SWISS-MODEL to search for homologous sequences of WW1 and WW2 part in YAP protein to highlight verteporfin-binding sites. Needle at EMBOSS is used for pairwise alignment between the sequences of known structure template with WW1 and WW2 domains.

### 2D Molecules selection

The 2D structure of drug molecule ligand used in docking (verteporfin) was obtained from PubChem (pubchem.ncbi. nlm.nih.gov) and the MarvinSketch software allowed us to have the 3D structures of these molecules.

### Docking analysis

The program AutoDock vina Version 31 (2010) 455-461 was used for docking [21] and the GUI AutoDockTools (ADT) version of 1 facilitated the preparation of files. This helped to transform the files in.pdb format and on.pdbqt format to determine the docking box on the target. The PyMOL was used for viewing.

### Alignment of WW1 and WW2 domains

The local alignment tool, Needle at EMBOSS (http://www.ebi.ac.uk/Tools/ emboss) was used for sequence alignment in this study.

## Results

**Figure 1:**
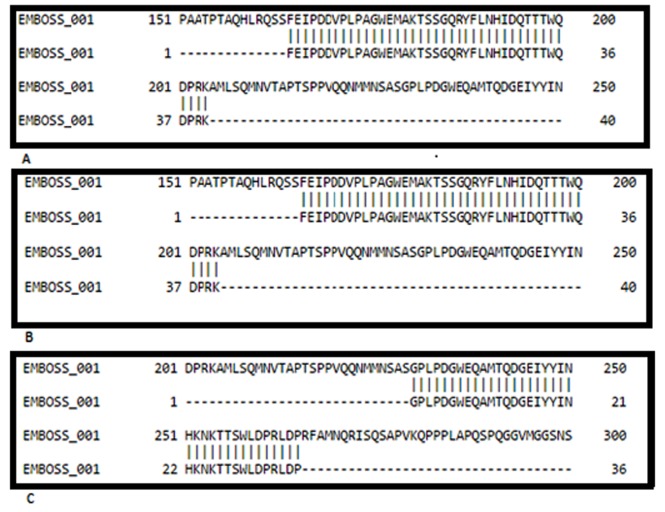
Pairwise sequence alignment between target (WW1 & WW2) and known structural templates (PDB ID: 1K9R, 1K9Q & 2LTV) using the global alignment tool Needle at EMBOSS.

The protein sequence of human YAP was retrieved from the Uniprot database to select templates. We used the Swiss model server to select known structural templates using the integrated BLAST service for model building. The sequence alignment between the WW1 and WW2 domains of human YAP and templates structures is given in Figure 1. The template structures selected in this analysis are (a) 1K9R (YAP65 WW domain in complex with Acetyl-PLPPY); (b) 1K9Q (YAP65 WW domain complex with N- (n-octyl) -GPPPY-NH2) and (c) 2LTV (YAP WW2 in complex with a peptide derived Smad7).

**Figure 2:**
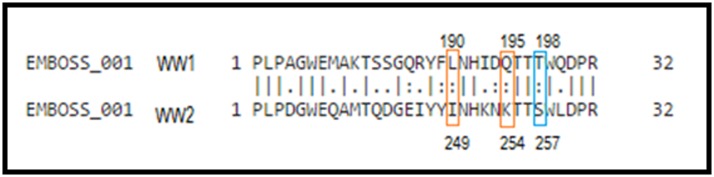
Alignment of WW1 and WW2 domains in YAP is shown using Needle at EMBOSS. W199, T197, H192, Y188 in WW1 are in WW2, L190, Q195 in WW1 replaced respectively by I249, K254 in WW2 (red frame). The WW2 residues involved in binding to verteporfin, W258, T256 are stored in WW1, the S257 of WW2 is replaced by T198 in WW1 and it is synonymous (blue frame).

Sequence alignment shows that 1K9Q and 1K9R are wild types of WW1, and 2LTV is the wild type of WW2. These templates are used for model building. PyMOL was used to remove bound ligands. The docking analysis shows the interactions between ligand and protein target. Several hydrogen bonds between verteporfin and amino acid residues of WW1 and WW2 domains of YAP were observed as given in Table 1. Thus, the interaction between verteporfin with the oncogenic transcription cofactor YAP is reported. The result of the first docking between this molecule and 1K9Q, it snaps into the triple sheet of β WW1 field with four hydrogen bonds, two with the T197 residue. The second docking of verteporfin with 1K9R, in this case, the molecule binds to both sides in the triple sheet β by five hydrogen bonds involving the residue T197 the β3 sheet and Y188 T182 on the sheet β1 giving stability to binding. The docking of the target protein with verteporfin 2LTV (WW2 domain) shows; there are six hydrogen bonds that allow the attachment to this site. The result of the alignment is given in Figure 2, and it shows a likeness of some residues in binding with the PPxY (W199, T197, H192, Y188 in WW1 are the same in WW2), others are similar (L190, Q195 in WW1 replaced respectively by I249, K254 in WW2). WW2 residues involved in binding to verteporfin, W258, T256 are conserved in WW1; the S257 of WW2 is replaced by threonine in WW1, which is synonymous.

## Discussion

The docking analysis shows verteporfin interactions with YAP WW1 domain by a network of hydrogen bonds. The docking with 1K9Q having four hydrogen bonds, of which 3 bonds with Y188 and T197 (one with Y188 and two with T197), residues interacting with the PPxY [14]. The docking with 1K9R shows 5 hydrogen bonds, 3 bonds with residues binding to PPxY, T197 and Y188. Thus, these two residues are essential for the binding of verteporfin with YAP WW1 domain. The association with the T182 that has no role with PPxY is likely to confer the stability of verteporfin with WW1 YAP complex.

In comparison with the binding of digitoxin to WW1 domain YAP, the latter binds to four amino acids, which are essential for binding PPxY (Y188, L190, T197 and W199) [16] while verteporfin binds with only Y188 and T197. So verteporfin can bind with two of the six amino acids essential for binding in a PPxY pattern. In vivo inhibition was known [17] suggesting verteporfin of structural alteration enhancing its specificity to WW1 of YAP. WW2 domain YAP is similar to WW1 domain, the docking with 2LTV domain WW2 shows that verteporfin forms 6 hydrogen bonds involving W258, T256 residues interacting with the PPxY [14] each with a bond, D264 and S257 by four bonds, giving more stability to the molecule. This did not lodge in the triple sheet β, as in the case of WW1.

Sequence alignment between WW1 and WW2 shows that residues W258 and T256 are conserved while S257 of WW2 is replaced by threonine in WW1 and it is synonymous (-OH side chain residue) (Figure 2). Alignment also shows that residues W199, T197, H192, Y188 involved in binding of the WW1 PPxY conserved in WW2 while L190 and Q195, respectively correspond to I249 and K254 which there are synonymous, suggesting the binding of verteporfin in WW2. Earlier reports have shown that proteins having PPxY can bind to both WW1 and WW2 domains of YAP with a preference for WW1. [15] This then suggests a modification of the structure of verteporfin for its binding to two domains YAP. A recent report [22] showed that verteporfin binds to TAED domain with only two hydrogen bonds. Data in this study reports the binding of the WW domain of verteporfin to YAP through the WW1 domain by four hydrogen bonds and to WW2 domain by six hydrogen bonds suggesting improved affinity to WW domains [22].

## Conclusion

The docking results of verteporfin with YAP WW1 and WW2 domains showed its ability to bind in the hydrophobic pocket and interact with residues involved in fixing PPxY implying its modulatory activity.

## Authors’ contribution

IK carried out the modeling and molecular docking studies and drafted the manuscript. WL and AB corrected the manuscript. AI edited the manuscript and has given final approval for publication.

## Conflict of interest

The authors report no conflicts of interest in this work.

## Figures and Tables

**Table 1:** Docked interaction analysis of verteporfin with WW domain of YAP

Ligand	Receptor (PDB ID)	Number of H-bonds	Active site residues	Number of interacting bonds	Bonds length in Å
Verteporfin	1K9Q	4	Y188	1	2.7
			T197	2	2.7 - 3.9
			T182	1	2
	1K9R	5	T197	2	3.0 - 3.5
			T182	2	2.8 - 2.0
			Y188	1	3.9
	2LTV	6	D264	3	3.6 - 3.6 - 2.4
			S257	1	2.8
			W258	1	2.4
			T256	1	3.6

Docked interaction analysis of verteporfin with WW domain of YAP (1K9Q, 1K9R and 2LTV).					

## Abbreviations

AMOTL1: Angiomotin-Like 1
LATS: Large Tumor Suppressor
FDA: Food and Drug Administration
PDB: Protein Data Bank
WW: Tryptophan-Tryptophan domain
YAP: Yes kinase-associated protein
TEAD: TEA domain-containing transcription
TAZ: transcriptional co-activator with PDZ-binding motif
PPxY: Proline-proline-x-Tyrosine.
